# Needs Assessment of Safe Medicines Management for Older People With Cognitive Disorders in Home Care: An Integrative Systematic Review

**DOI:** 10.3389/fneur.2021.694572

**Published:** 2021-09-03

**Authors:** Mojtaba Vaismoradi, Samira Behboudi-Gandevani, Stefan Lorenzl, Christiane Weck, Piret Paal

**Affiliations:** ^1^Faculty of Nursing and Health Sciences, Nord University, Bodø, Norway; ^2^Palliative Care, Paracelsus Medical University, Salzburg, Austria; ^3^Department of Neurology, Klinikum Agatharied, Hausham, Germany; ^4^WHO Collaborating Centre at the Institute for Nursing Science and Practice, Paracelsus Medical University, Salzburg, Austria

**Keywords:** aged, cognitive disorder, dementia, caregivers, family, home care services, medication therapy management, Alzheimer disease

## Abstract

**Background and Objectives:** The global trend of healthcare is to improve the quality and safety of care for older people with cognitive disorders in their own home. There is a need to identify how medicines management for these older people who are cared by their family caregivers can be safeguarded. This integrative systematic review aimed to perform the needs assessment of medicines management for older people with cognitive disorders who receive care from their family caregivers in their own home.

**Methods:** An integrative systematic review of the international literature was conducted to retrieve all original qualitative and quantitative studies that involved the family caregivers of older people with cognitive disorders in medicines management in their own home. MeSH terms and relevant keywords were used to search four online databases of PubMed (including Medline), Scopus, CINAHL, and Web of Science and to retrieve studies published up to March 2021. Data were extracted by two independent researchers, and the review process was informed by the Preferred Reporting Items for Systematic Reviews and Meta-Analyses (PRISMA). Given that selected studies were heterogeneous in terms of the methodological structure and research outcomes, a meta-analysis could not be performed. Therefore, narrative data analysis and knowledge synthesis were performed to report the review results.

**Results:** The search process led to retrieving 1,241 studies, of which 12 studies were selected for data analysis and knowledge synthesis. They involved 3,890 older people with cognitive disorders and 3,465 family caregivers. Their methodologies varied and included cohort, randomised controlled trial, cross-sectional studies, grounded theory, qualitative framework analysis, and thematic analysis. The pillars that supported safe medicines management with the participation of family caregivers in home care consisted of the interconnection between older people's needs, family caregivers' role, and collaboration of multidisciplinary healthcare professionals.

**Conclusion:** Medicines management for older people with cognitive disorders is complex and multidimensional. This systematic review provides a comprehensive image of the interconnection between factors influencing the safety of medicines management in home care. Considering that home-based medicines management is accompanied with stress and burden in family caregivers, multidisciplinary collaboration between healthcare professionals is essential along with the empowerment of family caregivers through education and support.

## Introduction

Cognitive disorders consist of several neurological conditions such as dementia and its most common subtype (70% of cases) Alzheimer's that influence the memory, cognition, thinking, behaviour, and functional ability to perform activities of daily livings. Age has been introduced as a strong risk factor for the development of cognitive and memory disorders (1). Given that 23% of the total global burden of diseases can be attributed to disorders among older people (≥60 years), neurological disorders are considered one of the leading contributors (6.6%) to disease burden in this age group (2).

Demographic transition has resulted in a significant increase in the elderly population, bringing degenerative neurological diseases including cognitive and memory disorders. Nowadays, 50 million people live with dementia worldwide, and the number will most likely rise to about 150 million by 2050 (3). As the matter of economic impact, the global estimation of the costs of dementia treatment and care has been US $957.56 billion in 2015, which will reach US $2.54 trillion in 2030 and US $9.12 trillion in 2050 (4). The devastating impact of cognitive and memory disorders on caregivers and family members should be added to this economic burden (3, 5). However, the burden of neurological disorders has been seriously underestimated by traditional epidemiological and health statistical methods that take into account only mortality rates rather than disability rates (6).

### Family Caregiving for Older People With Cognitive Disorders

Cognitive and memory disorders are multifactorial and complex healthcare conditions (7). According to the World Health Organisation (WHO) Ministerial Conference on Global Action Against Dementia in 2015, improvement of the quality of care delivered to these patients has been stated as a priority given its significance to the reduction of the global burden of these disorders in both individual and social levels (8). There is a huge gap in the workforce required to provide care to patients living with long-term illnesses and behavioural health issues (9). Therefore, development of community-based care initiatives, families' partnership, and consideration of institutional care as the last care resort have been emphasised for developing sustainable and high-quality care provision to these patients (10).

Family caregivers have the crucial role in the provision of long-term care and support to patients (11). Involvement of family members in designing and developing transitional care programs from hospital to own home and provision of support and education influences their commitment for collaboration (12, 13). Rapid and inappropriate transition of care including brief discharge plans, referral to the general physician or a primary caregiver without the full engagement of families have been shown to lead to insufficiencies in hospital-to-home transitions (14). New approaches to care planning for older people with cognitive disorders should include families and informal caregivers (15). However, the caregivers of patients with cognitive disorders often experience moderate or high levels of care burden that impacts their health, well-being, life satisfaction and resilience (16–18). Therefore, family caregivers need interaction and collaborative relationship with healthcare providers in the process of care transition to their own home leading to more patient-centred care (19, 20).

### Medicines Management in Home Care

Patients with cognitive disorders experience non-cognitive and psychotic symptoms, behavioural disturbances, and mood changes, which cause many challenges for both the patient and their caregivers (21). Poorer cognition and behavioural and psychological symptoms, impairments in performing activities of daily living, and burden of caregiving that accompany cognitive disorders increase the risk of admission to nursing homes (22). Therefore, the use of medications for symptoms' treatment among patients with cognitive disorders is associated with the improvement of functional and cognitive outcomes, fewer admission to nursing homes and hospitals, and the overall mortality (23, 24).

It has been shown that more than 40% of older people with cognitive disorders regularly use psychotropic medications such as antidepressants and cognitive enhancers (25). However, the rate of medication adherence among these older people ranges from 10.7 to 38% (26), which increases the risk of rehospitalisation after care transitions from hospital to own home (24). Therefore, family caregivers have the central position to perform home-based medicines management. The burden and distress of care in family caregivers should be reduced to improve the quality and safety of the medication process (11, 27, 28).

Previous reviews so far have concentrated on dementia home care by family caregivers and have not elaborated and specified the needs of family caregivers in home-based medicines management (29–31). Given the lack of integrated knowledge to inform the needs assessment of medicines management for older people with cognitive disorders who receive care from their family caregivers in their own home, this systematic review of international literature aimed to find the answer to the following question: What are the requirements of safe medicines management for older people with cognitive disorders by family caregivers in home care?

## Materials and Methods

### Design

The systematic review of international literature was carried out as an explicit method for collating and synthesising relevant empirical knowledge and giving a comprehensive answer to the research question (32). Since criteria for conducting meta-analysis or meta-synthesis could not be met on this research topic, an integrative review approach was chosen to include all empirical studies with qualitative and quantitative designs and to develop a comprehensive understanding of the healthcare problem through the creation of a connexion between numeric and narrative findings (33). The PICO statement was used for framing the review question, as follows: P: family caregivers of older people with cognitive disorders; I: medicines management in own home; C: requirement of medicines management identified by stakeholders; and O: safety of the medication process.

### Search Process

After the review protocol was developed and agreements on its details were reached by the authors, four online databases that mainly covered health sciences' literature were searched: PubMed (including Medline), Scopus, CINAHL, and Web of Science. It was aimed to retrieve all empirical studies without any limitation in the language and year of publication up to March 2021.

Inclusion criteria were all empirical studies with both qualitative and quantitative designs that involved the family caregivers of older patients with cognitive disorders in medicines management in own home and were published in peer-reviewed journal. On the other hand, reviews, commentaries, discussions, conference proceedings, letters to editor, and empirical studies on medicines management in acute and long-term healthcare settings were excluded.

The authors' previous experiences with conducting research on medicines management and the care process for older people with long-term mental health issues as well as a pilot search in general databases helped with identifying appropriate keywords. Also, a librarian in the affiliated university was approached to ensure the accuracy of keywords and database selections. Therefore, all probably relevant keywords and MeSH terms were identified and were used to build search phrases for conducting the search in titles, abstracts, and articles' contents using the Boolean method and the related operators (AND, OR). Cross-referencing from articles' bibliographies and a manual search in well-known journals that published relevant studies helped with improving the search coverage.

The titles and abstract of retrieved studies were carefully screened by the authors, and full texts were read to identify relevant studies to our review topic. However, decisions on the inclusion or exclusion of studies based on the inclusion criteria were through holding discussions by the authors.

### Quality Appraisal and Risk of Bias Assessment

Two authors (MV and SB-G) were made blind to studies' authors, journal name, and institution and independently evaluated the quality of each study using quality appraisal tools. They held discussions to share the evaluation results and to decide the inclusion and exclusion of each study.

The modified Consolidated Standards of Reporting Trials (CONSORT) was used for the appraisal of the methods and results sections of interventional studies. Studies with scores ≥70% of the highest score of the CONSORT checklist were judged as high quality, 40–70% as moderate quality, 20–40% as low quality, and <20% as very low quality (34).

The modified Newcastle–Ottawa Quality Assessment Scale was applied (35) for the quality appraisal of observational studies in terms of the selection of participants, comparability of the study, and assessment of outcomes. Scores above 6, 3–5, and below 3 were interpreted as high, moderate, and low quality, respectively.

The Critical Review Form—Qualitative Studies (Version 2.0) was used for assessing qualitative studies (36). It assessed studies in terms of purpose, justification of research, theoretical and philosophical perspectives for the design, method, sampling, data collection, data analysis, rigour, and conclusions and implications. Scores 1–6, 7–11, and 12–18 were interpreted as low, moderate, and high quality, respectively.

The ROBINS tool in non-randomised studies of interventions and observational studies was used for assessing the risk of bias (37), which has been recommended by the Cochrane (32). Five domains of (i) assessment of exposure, (ii) development of outcome of interest in case and controls, (iii) selection of cases, (iv) selection of controls, and (v) control of prognostic variable in cross-sectional studies; seven domains of (i) selection of exposed and non-exposed cohort, (ii) assessment of exposure, (iii) presence of the outcome of interest at the start of the study, (iv) control of prognostic variables, (v) assessment of the presence or absence of prognostic factors, (vi) assessment of outcome, and (vii) adequacy of follow-up for cohort studies; and also six domains of (i) bias in random sequence generation, (ii) bias in allocation concealment, (iii) bias in blinding of participants and personnel, (iv) bias in blinding of outcome assessment, (v) bias in incomplete outcome data, and (vi) bias in selective outcome reporting for interventional studies were used for the assessment. Accordingly, the authors' judgment for risk of bias was categorised as “low risk,” “high risk,” and “unclear risk” for interventional studies and high risk, low risk, and probability yes or no risk of bias for observational studies.

### Data Extraction and Knowledge Synthesis

Data from the selected studies were extracted independently by two authors (MV and SB-G) using an extraction table. The data were exported into the categories of author's name, publication year, country, design, sample size and setting, findings, and conclusion of home-based medicines management with the involvement of family caregivers.

The studies identified for this review had many variations in terms of aims, research structures, and methodological considerations. Therefore, a meta-analysis of findings could not be performed; and the review findings are presented narratively, which was informed by Preferred Reporting Items for Systematic Reviews and Meta-Analyses (PRISMA) statement (38).

## Results

### Search Results and Selection of Studies

**Figure 1:**
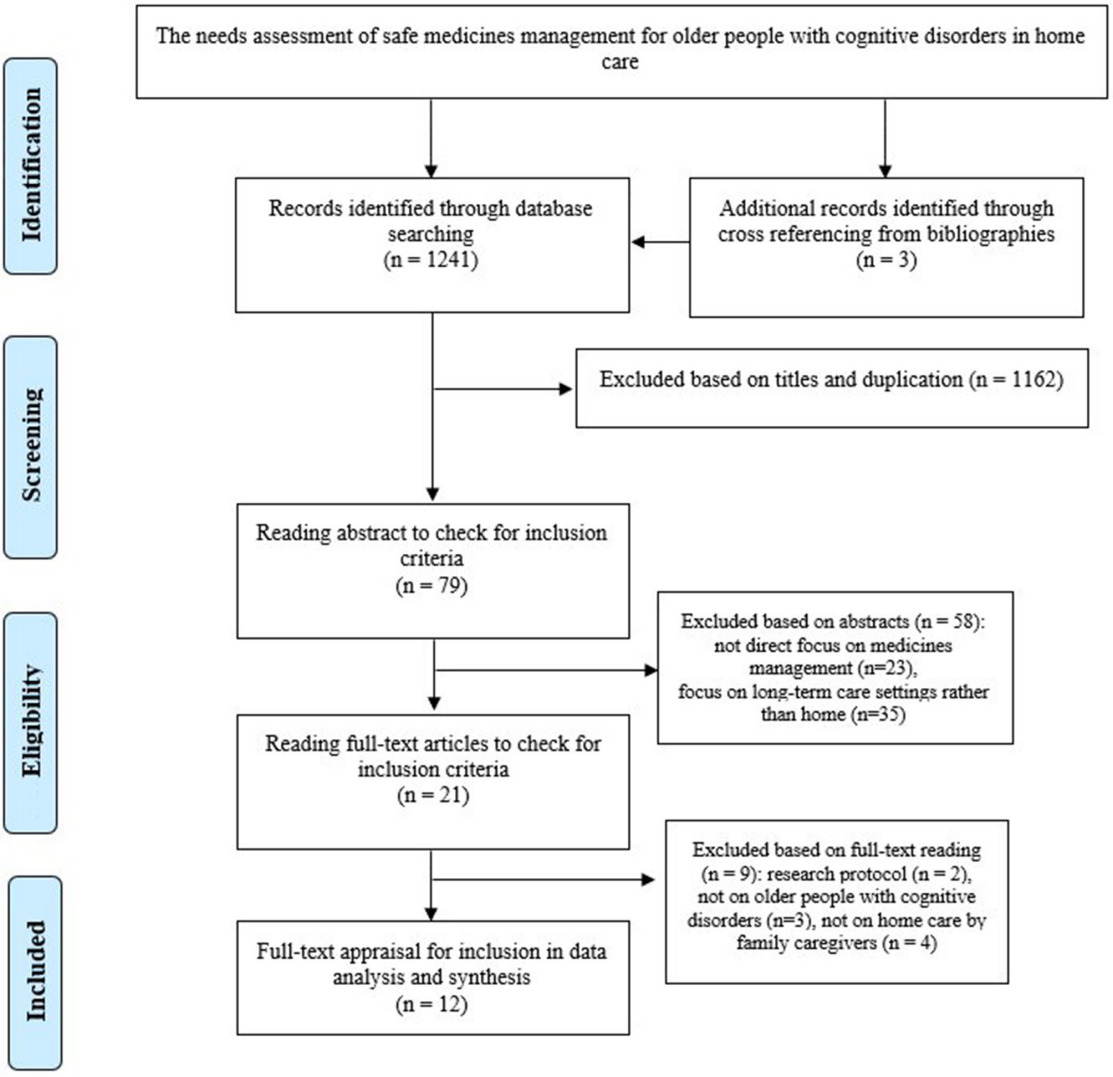
The Preferred Reporting Items for Systematic Reviews and Meta-Analyses (PRISMA).

The comprehensive search on the online databases and backtracking of references led to retrieving 1,241 studies (Table 1). After duplicates and irrelevant studies were deleted based on independent title screening and abstract reading by two authors (MV and SB-G), 21 studies were chosen for full text reading (Figure 1). They were carefully read, and their contents were checked against inclusion criteria, of which 12 studies fully met the criteria and were entered into full-text quality appraisals and risk of bias assessment.

**Table 1:** The result of search and article selection process.

**Search keywords**	**Databases**	**Total in each database**	**Selection based on title**	**Selection based on abstract**	**Selected based on full text**	**Selection based on quality appraisal and risk of bias assessment**
(medication OR drug OR medicines OR “medicines management” OR “medication management”) AND (old* OR elder* OR aged* OR senior*) AND (dementi* OR alzheimer* OR “cognitive impairment*”) AND (family OR spouse* OR partner* OR “family care*” OR “family nursing” OR caregiver* OR “informal care*” OR “non-professional care*” OR partner*) AND (home* OR domestic OR “home health nursing*” OR “home nursing*”)	PubMed (including Medline)	123	16	11	7	7
	Scopus	274	6	3	0	0
	CINAHL	409	5	0	0	0
	Web of Science	432	49	4	4	4
	*Backtracking references of selected articles*	3	3	3	1	1
	**Total**	1,241	79	21	12	12

### Quality Assessment and Risk and Bias Assessment

The full texts of 12 articles were assessed in terms of methodological quality and risk of bias. The quality assessment of the included studies has been presented in Supplementary Tables 1–4 and Supplementary Figures 1–3. Nine studies were classified as high quality (39–47) and three as moderate quality (48–50), and no study had low quality.

The studies mostly were judged as having low risk of bias for the evaluated domains (Supplementary Figures 1–3). Accordingly, all cross-sectional studies (40, 41, 48, 50) had a low risk of bias in the assessment of exposure and development of outcome of interest. However, two-thirds of them had probability high risk of bias in selection of case and controls, and half of them had high risk of bias in control of prognostic variable.

There was one cohort study (49) that had low risk of bias for adequacy of follow-up of cohorts, assessment of outcome and exposure, and assessment of the presence or absence of prognostic factors. However, it had high risk or probable high risk of bias in the selection of exposed and non-exposed cohorts, control of prognostic variable, and presence of outcome at start of study.

In interventional studies, all studies (39, 42, 46) had a low risk of bias in the reporting of selective outcomes, incomplete outcome data, and random sequence generation. However, two-thirds of them had a high risk or unclear risk of bias in the blinding of personnel, participants, and outcome assessment. In addition, all of them had an unclear risk in the allocation concealment.

Therefore, all studies (*n* = 12) were included in the data analysis and knowledge synthesis given their acceptable methodological structure and relevance to our review topic.

### Characteristics of Selected Studies

The general characteristics of the selected studies have been presented in Table 2. They were published between 2006 and 2017, indicating that the search process encompassed a decade research on this topic. They involved 3,890 older people with cognitive disorders and 3,465 family caregivers.

**Table 2:** General characteristics of selected studies included to data analysis and knowledge synthesis.

**Author, year, country**	**Aim**	**Methods**	**Sample and settings**	**Outcome measurement**	**Main finding**	**Conclusion**	**Quality appraisal**
Cotrell et al. (2006), USA	To investigate the cognitive status of patients, skills for medicines management, adherence to medications, and amount of help received from family caregiver	Cross-sectional	27 (male/female) older people (>65 years) with Alzheimer's and 20 (male/female) healthy older people, dyad caregivers as spouse, children, and other relatives in home care	Complexity of the medication regimen, behaviour of adherence using pill counts, predicated adherence, medicines management tasks, prediction of task, awareness discrepancy	Acceptable level of adherence to medications but ineffectiveness of some strategies by family caregivers to ensure sufficient adherence	The need to adopt additional strategies by family caregivers for medicines management	Moderate
Brodaty et al. (2009), Australia	To examine the effect of a 3-month psychosocial counselling intervention focused on symptoms, emergencies, and managing difficult behaviours for the family caregivers of older people with Alzheimer's taking donepezil (5–10 mg daily) on their admission to the nursing home and mortality	Randomised controlled trial: 2 years' treatment and up to 8.5 years' follow-up	155 intervention and control (male/female) older people (>70 years) with Alzheimer's and their (male/female) family caregivers (>70 years) as dyad in home care in Australia, the United Kingdom, and the United States	Time to admission to the nursing home and death, concurrent medications and related adverse events, the older people's physical health	Similar nursing home placement and mortality in the intervention group, but Australians admitted earlier	Variations in healthcare, nursing home systems, and affordability of care influence on admission to the nursing home more than other factors	High
Lau et al. (2010), USA	To investigate the association between medication use and potentially inappropriate medication use among older people with and without dementia	Retrospective cross-sectional	4,518 (male/female) older people (≥65 years) with (*n* = 2,665) and without dementia (*n* = 1,853) living with the family as spouse, partner, family, relative, or friend	Potentially inappropriate medication use, as those medications should be avoided among elderly people, number of prescription medications used excluding *pro re nata* (PRN) and over the counter (OTC)	Increased risk of inappropriate medication use and polypharmacy	The need to evaluate the necessity and appropriateness of medications in home care to reduce the risk of admission to the nursing home	High
Erlen et al. (2013), USA	To describe the characteristics of family caregiving for medicines management in home care for older people with impaired cognition	Cross-sectional	91 dyads (male/female) of older people (80 years)—family caregivers (67 years) who were spouse/non-spouse	Sociodemographic and health-related characteristics, health literacy, working memory, source of stress, older patients' aggressive/disruptive behaviours and caregiver's reactions, self-confidence, depressive symptoms, perception about problem solving, social support resources, medicines management	Caregivers' demographic characteristics, cognitive abilities, psychological condition, and perception influence caregiving in home	Significance of the family caregiver's demographic and health-related characteristics in medicines management in home care	High
Fiss et al. (2013), Germany	To investigate the frequency of potentially inappropriate medications taken by older people with dementia cared by family caregivers in comparison with healthy older people	Cohort	342 older people (≥80 years) consisting of 111 (female/male) with dementia and 231 (female/male) healthy ones	Sociodemographic and health-related variables, cognitive impairment, home medicines review, identification of potentially inappropriate medications for older people, and potentially inappropriate for those with dementia	20% older people with dementia had potentially inappropriate medications; number of medications (1–4) was a risk factor for it	Receiving <5 medications and support in home care protected against potentially inappropriate medications. Systematic medication review in home care should be established	Moderate
While et al. (2013), Australia	To explore the perspectives of older people with dementia and their family carers regarding medicines management and compare them with those of healthy older people	Grounded theory	8 older people with dementia and 9 family caregivers (spouse and child)	Self-medicines management of prescribed and non-prescribed medications in home care and the family member support	Life routines and established caring strategies can enable older people with dementia to perform self-medicines management. Family members can support independence in medicines management	Family caregivers are responsible for medication safety in home care, but their stress and the burden of care should also be considered	High
Poland et al. (2014), UK	To identify the perspectives of family caregivers of older people with dementia about medication management in home care	Thematic analysis	9 family caregivers (spouse and child)	Priorities, benefits, and side effects of medications, adherence, prescription and administration, medication review, communication with healthcare staff	Use and administration of medications, communication issues, responsibility and accountability, medications' risks, and benefits	Lack of appropriate support for medicines management and need to empower them to directly become involved in care	High
Smith et al. (2015), UK	To explore the experiences of family caregivers about how to make medicines management more responsive to the needs of older people with dementia	Framework analysis	9 (male/female, 45–86 years) family caregivers (spouse and child) and 5 older people with dementia (male/female, 81–93 years)	Activities related to medicines management and problems experienced by caregivers	Complexity of care and decision making for medicines management, medication supplies, adherence to the regimen, and access to healthcare providers, obtaining information and advice, older people's autonomy	Need for strategies to reduce burden of care through training and support	High
Thyrian et al. (2016), Germany	To analyse the various aspects of dementia care including medicines management for older people in own home after receiving multi-professional and multimodal individualised care to improve dementia care at home	Cluster randomised controlled trial	516 older people (≥70 years) and their family caregivers as dyads: intervention (*n* = 348), control (168)	Medication review on antidementia drugs (donepezil, rivastigmine, galantamine, memantine, and their combinations) and antidepressants, as well as OTC: compliance, adverse effects, administration of medications, potentially inappropriate medications as the risks of adverse events outweigh benefits	About 26% received antidementia medications and 14% received antidepressants	Complexity and multivariate identity of home care and	High
Lingler et al. (2016), USA	To examine the effect of a problem-solving intervention for improving medicines management among the caregivers of older people with dementia	Randomised controlled trial	76 older people and their family caregivers (spouse and child) as dyads: intervention (*n* = 37) and control (*n* = 39)	Medicines management practise and deficiencies	Reduction of medication problems 2 months after the intervention	Effectiveness of raising awareness of significance of medication safety	High
Maidment et al. (2017), UK	To explore the key challenges of medicines management from the perspectives of older people with dementia and their family caregivers in home care	Framework analysis	11 family caregivers (male/female), 4 older people with dementia (male), 16 healthcare providers (male/female)	Practical issues and challenges of medicines management	Responsibility for medicines management, emotional burden of care, obtaining support	Need for coordinated and continuous support for family caregivers, medication review, improving the role of community pharmacists in home care initiatives	High
Wucherer et al. (2017), Germany	To identify the prevalence and type of drug-related problems and associated factors among older people with dementia in home care after the implementation of collaborative dementia care management	Cross-sectional	446 (>79 years) older people with dementia (male/female), family caregivers (*n* = not specified)	Medication assessment: medication history (prescription and OTC), compliance, adverse events, administration of medication	1,077 drug-related problems were found; 93% had at least one problem with administration and compliance, drug interactions, medication selection	Association between the number of medications and medication problems	Moderate

Four studies were conducted in the United States (40–42, 48), three studies in the United Kingdom (43–45), three studies in Germany (46, 49, 50), and two studies in Australia (39, 47).

The studies had variations in methodologies including cross-sectional studies (40, 41, 48, 50), randomised clinical trials (39, 42, 46), cohort (49), and qualitative studies (43–45, 47).

The studies aimed to assess for skills and adherence to home-based medicines management (40, 43–48), interventions to support family caregivers (39, 42), and inappropriate medication use and drug-related problems (41, 49, 50).

### Needs Assessment of Safe Medicines Management in Home Care

The older people participating in the studies suffered from dementia and had various levels of cognitive impairment from mild to severe. Also, the mean number of medications taken by them in home care was between a minimum of 4.9 and a maximum of 10, indicating over-medication and polypharmacy, respectively. Overall, their adherence to medications was low; and therefore, all older people needed and received support for medicines management from family caregivers in home care. Family caregivers were taken as responsible and were involved in all interventions related to home-based medicines management including dispensing, preparation, administration, follow-up, and monitoring the effects and side effects of medications (Table 3).

**Table 3:** Medicines management and the need of older people with cognitive disorders to receive support in their own home.

**References**	**Level of cognitive impairment**	**Mean number of medications taken by older people**	**Adherence to medications**	**Older people's need to receive help for medicines management from family caregiver**	**Areas of need to support for home-based medicines management by family caregivers**
Cotrell et al. (48)	Mild–moderate	Not specified	17–100%	Yes	Checking and setting up pill box, timing, dosing, naming and preparation of medications, administration of medications
Brodaty et al. (39)	Moderate–moderately severe	Not specified	Not specified	Yes	Dosage, preparation and administration of medications, assessing effectiveness of medications, concurrent medications use, alcohol–medication interaction, adverse events
Lau et al. (41)	Very mild–severe	4.9	Not specified	Yes	Not specified
Erlen et al. (40)	Moderate	10	Acceptable level	Yes	Supply, storage, timing, being reminded to take medications, mixing, administration of medications
Fiss et al. (49)	Mild and suspicious	6.8	No	Yes	Preparation and administration of medication
While et al. (47)	Not specified	Not specified	Yes	Yes	Filling dosette box, dosage, supply, administration of medications, monitoring side effects, tracking medications and renewal
Poland et al. (44)	Not specified	Not specified	Not specified	Yes	Preparation, mixing, medication administration based on the older people's need, communicating medication-related issues to healthcare providers, deciding on the discontinuation of medications, monitoring effects and side effects
Smith et al. (45)	Various	7	Low level	Yes	Supply, refill, filling dosage box, timing, monitoring effects and side effects, communicating with healthcare providers
Thyrian et al. (46)	Mild	Not specified	Not specified	Yes	Preparation and administration of medication
Lingler et al. (42)	Mild	10	Low	Yes	Pharmacy pickup, storage, pillbox, medication administration (OTC) over the counter medications, receiving support from local pharmacist, backup list for someone else to administer medications, discarding discontinued medications, changing medications
Maidment et al. (43)	Not specified	Not specified	Low	Yes	Supply, timing, administration of medication, deciding on the discontinuation of medications
Wucherer et al. (50)	Mild–severe	>5	Low	Yes	Storage, timing, medication list preparation, administration

### Older People's Dependence on Family Caregiving in Their Own Home

Family caregivers were mentioned to be in the best position to accurately assess the ability and performance of older people with cognitive impairment to manage medications and to ensure that the safe level of adherence to the medication regimen was achieved (45, 48, 49). They tried to improve older people's independence in medicines management as much as possible and enhance their confidence in self-care. Older people tried to learn about medications and remember regimen using the visual recognition of medications, linking medications' taking to life routines, memory aids as board notices, and dose administration aids (47). However, they were unable to perform the medication process safely (45, 48, 49). They showed worse functions in medicines management tasks, including timing, dosing, preparation and naming medications, and medication intake, due to forgetfulness and administration of medications (48, 49). They also relied heavily on their family caregivers to regularly supply their medications given that no such a care option was available by healthcare providers in home care (47). Therefore, family caregivers were on the duty of older people care between 16 and 24 h a day on average for the provision of support (40), which influenced the quality and safety of the medication process. The greater the level of cognitive impairment and awareness deficit, the greater the support for the preparation and administration of medications was needed. Consequently, those older people who received more support in their activities of daily living from their family caregivers had greater adherence to medications than those who received less support (48).

### Family Caregivers' Concerns and Strategies for Medicines Management

Medicines management was mentioned as a complex process that required adopting routines. Family caregivers had no structurally defined role and did not receive education and support to perform medicines management tasks. Insufficient problem-solving skills, poor cognitive and memory function, and co-morbidities in family caregivers who had to manage their own medications at the same time enhanced the burden of care and the possibility of medication errors (40, 47). Also, caregivers' age was associated with deficiency in medicines management in terms of knowledge of medications and how to carry out the medication process (40). Additionally, the emotional burden of care encompassed having the obligation to take responsibility of the medication process for someone else and prioritising others' health on their own health (43). In this respect, decision making by family caregivers on the administration of sleep medications to older people to promote rest in family caregivers created an ethical challenge as it counterposed the health needs of family caregivers and those of older people who needed advocacy (44).

Taking medication at different times of the day and supply of medications were main challenges from family members' perspectives (43). Family caregivers were responsible for monitoring supplies from various prescriptions and timely refilling medications. Therefore, changes in prescriptions were added to the burden of care regarding taking correct medications (45).

Medication administration also enhanced their anxiety and care burden given the possibility of error during filling the dosette box. They tried to prevent medication errors by undertaking the task when they felt fresh and had more readiness to perform complex caring tasks (45, 47). Missed doses because of older people's reluctance to take medications were another concern. To overcome this barrier, they tried to inform older people and share information with them to involve them in decision making regarding medications to feel control over their own medications (45). However, adherence was difficult, as not all older people could understand the significance of taking medications, because of the complexity of regimens and not all medications taken regularly had a visible impact on their symptoms (43, 47). Explaining the reason for the administration of medications for relieving visible signs and symptoms reduced older people's resistance to adherence (44, 47). Regular and frequent visits and reminders *via* phone calls by those family caregivers who did not live with older people ensured that medications were taken timely (45).

Family caregivers felt frustration over the ineffectiveness of medications on improving the behaviour and memory of older people (43). They monitored the effectiveness and side effects of medications through observing older people's behaviour such as being tired and accordingly made judgments (45). They also were worried about taking over the tasks of medicines management and communicating routines to other family members or healthcare providers in emergency situations and hospitalisation. They used their mobile phones and created a backup of the list of medications and asked another family member to save it (47).

### Medicines Management Issues in Home Care

Rapid changes in cognitive abilities, complexity of medications, side effects of medications, and transition of care to the hospital and then back to own home hindered family caregivers in undertaking home-based medicines management safely (47). Also, insufficient use (21%) of healthcare services such as physiotherapist, occupational therapist, and speech therapy indicated inadequate or limited access to such services, which in turn led to overreliance on medication use for relieving health issues (46). About 55% of caregivers made at least one medication error, and an average of three deficiencies in medication was reported by 92.3% of them. Medication reconciliation identified 56% medication errors in terms of wrong time, forgetting to take the medication, losing pills, refilling prescriptions, mixing medications inappropriately, discontinuing medications without consultation, not taking medication on an empty stomach, and dumping pills into water (40). In another study, administration and compliance issues (60%), all potential drug-related interactions (17%), inappropriate selection (15%), dosage (6%), adverse drug events (2.5%), inappropriate time of application (40%), inappropriate combinations and interactions with moderate severity (35%), lack or outdated medication list (25%), inappropriate medication (23%), forgetting to take medications (18%), inadequate storage of medications (44%), and inappropriate storage as poor traceability, being exposed to moisture or light, and being scattered around the house (41%) were reported (50).

In addition to donepezil and other cognitive-enhancing drugs such as cholinesterase inhibitors and anticholinergic drugs, older people took many medications for cardiovascular, nervous, digestive, and respiratory disorders; osteoporosis; joint pain; and mental and psychiatric health issues (45, 46, 49, 50). Taking more medications was associated with more medication deficiencies and errors in home care (40). Therefore, over-medication as taking many medications at the same time and polypharmacy as taking more than five medications increased the risk of potentially inappropriate medications use and were considered safety concerns. They potentially worsened behavioural and psychological symptoms and made the family caregivers worried about medications' effectiveness and side effects (41, 47, 49, 50). Increasing the total number of medications increased the risk of potentially inappropriate medication use, as follows: five to six medications, 6.44 times; and seven to eight medications, 12.6 times (41).

The presence of co-morbidities including hypertension, incontinence, depression, and anxiety in these older people increased potentially inappropriate medication use, as 15% of older people had at least one potentially inappropriate medication use with the following medications: oral oestrogens (14%), muscle relaxants and antispasmodics (14%), fluoxetine (13%), short-acting nifedipine (11%), and doxazosin (7%) (41). In another study by Thyrian et al. (46), about 19.3% had one, 2.3% two, and 0.2% three potentially inappropriate medications. In the study by Fiss et al. (49), 27% received potentially inappropriate medications, and 20% received medications that were contraindicated in these patients including antidepressants (mostly amitriptyline), hypnotics (zolpidem), and anxiolytics (diazepam). In the study of Wucherer et al. (50), 92.8% had at least one drug-related problem, 64% had one to three drug-related problems, and 27% had four to seven drug-related problems. Also, 8% of older people received medications with a high dosage, and 6% reported adverse drug events related to a prescribed medication. The most frequently prescribed potentially inappropriate medications were antidepressants, benzodiazepines, and analgesics. On the other hand, the appropriate use of Fybogel as a laxative for relieving constipation as a minor health issue reduced physical and emotional distress among older people (44).

Both polypharmacy and potentially inappropriate medication use enhanced the risk of falls (72%) and adverse drug effects considering that these older people were sensitive to cognitive impairments induced by medications including confusion, nightmare, agitation, and depression, which enhanced the risk of admission to the nursing home (41, 49, 50). Given the cost of admission to nursing homes and the reported survival rate in there in the United Kingdom and Australia, the safety of home care in the hands of family caregivers depended on care supervision by healthcare professionals to monitor the effects and side effects of medications and help with resolving medication-related issues that were beyond the abilities of family caregivers (39).

### Support for Medicines Management in Home Care

Listening to family caregivers' concerns and provision of verbal and written information at their understanding were important, but more assistance with problem solving for managing medications in home care was required (40, 43). Physicians, pharmacists, nurses, older people, and family caregivers should coordinate medication-related care, as it created the feelings of safety, confidence, and assurance in home care (46, 47). Coordinated actions from various healthcare providers such as compliance packs prepared by pharmacists and support by nurses with *pro re nata* (PRN) medications specifically were needed (43). Family caregivers needed a structured list to keep track of medications when renewal was needed, and authorisation of prescription could be granted *via* phone calls. Inconsistencies in collaboration by healthcare providers led to frustration and stress (44, 45, 47). Also, absence of the medication list contributed to the high number of administration and compliance problems (50). For example, home visits by the nurse or social worker along with telephone calls to support the family caregivers' role in medicines management in terms of preparation, administration, and follow-up reduced the number of problems and deficiencies in medicines management in terms of dropping or losing pills, forgetting to take medications, dosage issues, and wrong times of medication administration (42).

Medication review by healthcare professionals was required to reduce the complexity of the medication regimen, leading to changes in medications and replacing them with those that could be administered with fewer doses and administering times, which consequently could improve adherence (43). A home-based medication review on prescribed and over-the-counter (OTC) medications not only improved medication compliance but also enhanced appropriate storage of medications (50). It should go beyond asking the patient about taking and not taking medications and should encompass dosage, effects, and side effects of medications (40, 43, 50). It could help with rectifying the misperception in family caregivers who deprescribe and stop medication could endanger the quality of life of loved ones (43, 44). The result also should be shared with other healthcare providers to enable care coordination and reduce the burden of sharing complex information by families and older people (43).

Considering the effect of progression of cognitive impairment on learning and developing skills for the medication process, family caregivers should be involved in the hospital discharge plan and be informed of changes in the medication regimen. Family caregivers could influence older people's beliefs and preferences to take medications and adhere to the medication process and were able to monitor and report medications' side effects (45, 47).

A supportive carer–healthcare professional relationship was needed to improve their knowledge about medications and enhance their power and feeling of control. Family caregivers felt despair in communicating medication-related issues and getting support from healthcare providers, as they felt that healthcare providers put all responsibility of care on their shoulder and did not advise about the practical aspects of medicines management (44, 47). Given that older people with cognitive impairment were unable to communicate their needs, family caregivers wanted to learn about identifying older people's needs to medications through observations and interpretation of behavioural clues (44).

Knowledge of medications was important; and family caregivers preferred to discuss with healthcare providers about rationale for prescription and the balance between the benefits, side effects, and harms of medications. They needed to be empowered to be able to monitor and report the effect of the medication regimen, side effects, and adverse drug reactions (44, 47). The role of family caregivers in the control of medication use and making decisions on their continuance of use was unclear, as no healthcare provider was accessible to monitor medications for pain relief, hypertension, osteoporosis, diabetes, and eye problems as well as PRN medications (44). Family caregivers proactively sought information about medications through reading packages, searching the internet, and making phone calls to healthcare providers regarding the type of medication, dosage, and related side effects (45). However, medication packaging was not helpful given difficulties in understanding and the multiple use of medications. Information should be simplified based on culture and language abilities and be interpreted to become appropriate to information-seeking needs particularly for the most common side effects and how to make decisions on them in the absence of access to expert knowledge (43–45).

**Figure 2:**
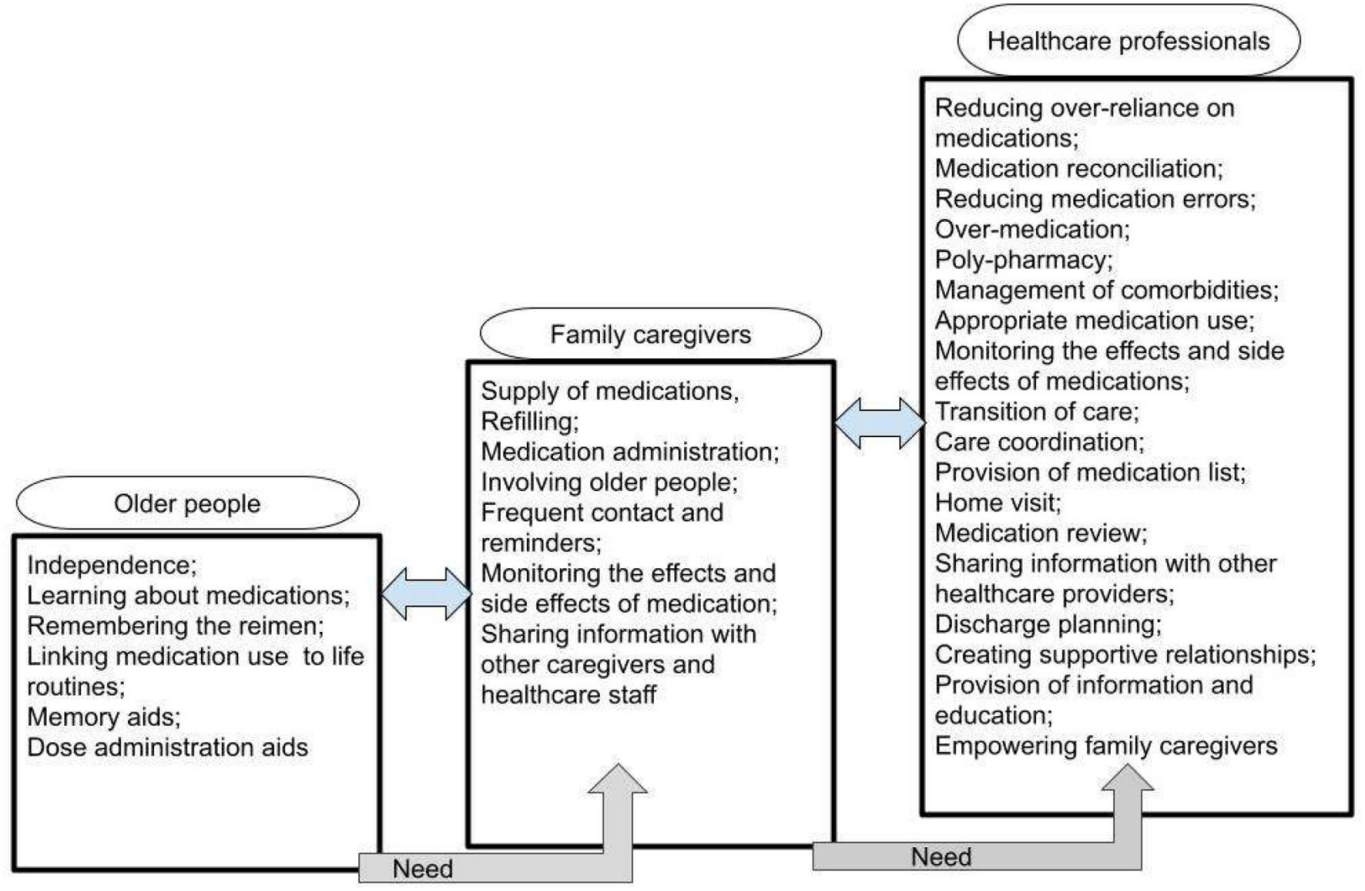
The needs assessment of safe medicines management for older people with cognitive disorders who are cared by their family caregivers in own home.

A summary of the review findings regarding the needs assessment of safe medicines management for older people with cognitive disorders who are cared by their family caregivers in their own home is presented in Figure 2.

## Discussion

This systematic review with an integrative approach helped with removing the gap of knowledge and enhanced our understanding of needs assessment of home-based medicines management for older people with cognitive disorders who were cared by their family caregivers. The review findings indicated the areas of needs of older people with cognitive disorders and their family caregivers in home care and what the role of healthcare professionals could be to help with safeguarding medicines management.

Older people with cognitive disorders preferred to remain independent as much as possible and to gain more control over their own medications. Family caregivers complied with older people's preferences, but progression of the disease and memory issues were barriers to retain independence. Therefore, the burden of medicines management was put on the shoulder of family caregivers who themselves needed support to manage medications for their own underlying health conditions and to reduce care stress. Collaborative strategies for medication management depend on the disease stage, and physical and mental capacity of older people as well as collaboration inputs by family caregivers (51). According to the statement by the United Nations (UN) and the WHO, facilitation of access to rehabilitation and palliative care is considered an ethical responsibility of healthcare systems. Also, healthcare professionals have the duty to alleviate pain and suffering among older people with physical, psychosocial, or spiritual sources irrespective of the curability of the disease (52). Any intervention aiming at the reduction of frailty among older people enhances benefits for individuals, families, and the society as they experience less cognitive or functional decline and have lower mortality rates (53). Family caregivers take different roles during the care process as caregivers, welfare enhancers, facilitators, apprentices, and minimisers/managers of suffering. They carry out many tasks and are responsible for the continuity of care and making decisions at the end of life (54). In the caregiving relationship, burden, resilience, needs, and rewards are interrelated (55). Female and male caregivers take on different tasks, which come with gender-specific care burden and health-related concerns. Sex- and gender-based analyses regarding caregiver's burden are limited. In terms of preparedness, being female and cohabiting with the patient have been associated with a higher level of preparedness to take over the caregiver's role (56). All caregivers achieve lower scores on physical and mental health measures than the general population (57). Studies assessing caregiver's burden have found higher burden or care-related distress among female caregivers as well as significantly higher levels of depression in female caregivers compared with their male counterparts. In terms of mental health, women report two times higher depression, but there have been suggestions within the international literature that men's experiences of depression may manifest with symptoms that are not currently included in traditional depression scales. In terms of physical health, female caregivers experience better sleep quality and significantly less co-morbidity, but male caregivers demonstrate biomarkers for increased thrombosis and inflammation risk (58). Prolonged grief disorder is predicted by the poor physical and mental health status before bereavement (57). Caregiver's health impacts the patient's quality of life and dying. Caregiver's capacity and preparedness for the provision of care can ensure quality of life, care, and death for older patients with memory disorders. Caregiver's fair-to-poor health status can predict non-elective hospital visits as well as hospital death (59).

Despite the family caregivers' crucial role, safety of the medication process could not be fully preserved, and medication errors and non-adherence to the medication regimen were reported in home care. The full compliance with the safety initiatives of home-based medicines management needed the support of healthcare professionals. Healthcare professionals should reduce over-reliance on medications; prevent medication errors; manage over-medication, polypharmacy, and inappropriate medication use; and monitor the effects and side effects of medications. Safety of medicines management in home care required that healthcare professionals coordinate discharge planning and care transition, attend home visits, and share information between other healthcare providers involved in home care. Moreover, a supportive and professional family caregiver–healthcare relationship with an emphasis on considering family caregivers' concerns, their education, and empowerment to safely perform the medication process was needed. The accepted perspective is that older people with cognitive impairment living in the community need coordinated and flexible care process (60–62). An early integration of holistic palliative care approaches that encompass medicines management initiatives into home care should be included from the beginning of the illness (63, 64). *The Lancet*'s call for action specifies “as the world population ages, comorbidity also increases, a shift from a health system centred in medical specialties to person-centred care is required.” This call also includes the provision of education and support to family caregivers, whose role in providing the best care for people with memory disorders should not be overlooked (65).

The heterogeneity of the studies included in this systematic review in terms of methods and aims hindered conducting a meta-analysis. Also, a few studies were retrieved during the search process, indicating the insufficient number of empirical studies. Nevertheless, this review provides an overview of international knowledge about home-based medicines management for older people with cognitive disorders by their family caregivers and aspects that should be investigated in future studies. Clinical trials are needed to improve our understanding of the effect of home-based medicines management interventions with the participation of family members on the quality and safety of care. Equally significant are the realist evaluations of any medicines management initiatives or educational activities, which provide a framework for understanding how the context and underlying mechanisms affect the pattern and outcome of the selected intervention.

## Conclusion

This integrative systematic review demonstrated that medicines management in home care was systematically overlooked adding to caregiver's burden and endangering the safety of older people. Family caregivers' abilities in the provision of care to older people with cognitive impairment could not cover all aspects of home-based medicines management. Therefore, the burden of medicines management in home care can be reduced through sharing the responsibility of safeguarding medicines management between family caregivers and healthcare professionals to be able to safely respond to older people's care needs.

Healthcare professions involved in home care are expected to proactively assess and meet older people's needs for safe medicines management in home care and relieve the great amount of stress and burden experienced by family caregivers. Consideration of family caregivers' concerns, continuous communication with them and provision of information about medications, discussion about medicines management strategies, empowerment of older people with memory disorders and their caregivers through education, and multidisciplinary collaboration have been emphasised.

## Data Availability Statement

The original contributions presented in the study are included in the article/Supplementary Material, further inquiries can be directed to the corresponding author.

## Author Contributions

MV and PP: conceptualisation. MV, SB-G, and PP: data curation, formal analysis, investigation, and methodology. MV and SB-G: project administration, resources, and software. MV, SB-G, SL, CW, and PP: writing—original draught, writing—review, and editing. All authors have read and agreed to the published version of the manuscript.

## Conflict of Interest

The authors declare that the research was conducted in the absence of any commercial or financial relationships that could be construed as a potential conflict of interest.

## Publisher's Note

All claims expressed in this article are solely those of the authors and do not necessarily represent those of their affiliated organizations, or those of the publisher, the editors and the reviewers. Any product that may be evaluated in this article, or claim that may be made by its manufacturer, is not guaranteed or endorsed by the publisher.
