# Supplementary material for: Needs Assessment of Safe Medicines Management for Older People With Cognitive Disorders in Home Care: An Integrative Systematic Review
Source: Front Neurol. 2021 Sep 3;12:694572. doi: 10.3389/fneur.2021.694572 (PMC8446192; doi:10.3389/fneur.2021.694572)
Supplement: Supplementary file 1 [file Data_Sheet_1.docx]

Supplementary table 1. Quality assessment of included studies using the CONSORT Assessment Scale for interventional studies.

| Author | Methods | | | | | | | | | | | | | | | | |
| --- | --- | --- | --- | --- | --- | --- | --- | --- | --- | --- | --- | --- | --- | --- | --- | --- | --- |
|  | Trial design | | Participants | | Interventions | Outcomes | | Sample size | | Randomization | | Allocation concealment mechanism | Implementation | Blinding | | Statistical methods | |
|  | a | b | a | b |  | a | b | a | b | a | b |  |  | a | b | a | b |
| Brodaty et al., 2009 (39) | - | * | * | * | * | * | * | - | * | * | - | - | * | * | * | * | * |
| Thyrian et al ., 2016 (46) | - | * | * | * | * | * | * | - | * | * | * | - | - | - | - | * | * |
| Lingler et al., 2016 (42) | - | * | * | * | * | * | * | - | * | * | * | - | - | - | - | * | * |

| Author | Results | | | | | | | | | | Total | Quality |
| --- | --- | --- | --- | --- | --- | --- | --- | --- | --- | --- | --- | --- |
|  | Participant flow (a diagram is strongly recommended) | | Recruitment | | Baseline data | Numbers analyzed | Outcomes and estimation | | Ancillary analyses | Harms |  |  |
|  | a | b | a | b |  |  | a | b |  |  |  |  |
| Brodaty et al., 2009 (39) | * | * | * | * | * | * | * | * | * | * | 23 | High |
| Thyrian et al., 2016 (46) | * | * | * | * | * | * | * | * | * | * | 21 | High |
| Lingler et al., 2016 (42) | * | * | * | * | * | * | * | * | * | * | 21 | High |

Supplementary table 2**.** Quality assessment of the selected studies using the Newcastle–Ottawa Quality Assessment Scale for cohort studies.

| Selection | | | | | Comparability | Outcome | | | Total score | Quality |
| --- | --- | --- | --- | --- | --- | --- | --- | --- | --- | --- |
| Author, year | Representativeness of the exposed cohort | Selection of the non-exposed cohort | Ascertainment of exposure | No outcome of interest at the start of the study | A: study controls for age and/or BMI  B: study controls for other confounders | A: doctor’s diagnosis OR objective measurements  B: parent/self-reported doctor’s diagnosis OR use of medication | Follow-up long enough for outcomes | Adequacy of follow up of cohorts |  |  |
| Fiss et al., 2013 (49) | - | - | * | - | - | * | * | * | 4* | Moderate |

Supplementary table 3**.** Quality assessment of the selected studies using the Newcastle–Ottawa Quality Assessment Scale for cross-sectional studies.

|  | Selection | | | | Comparability | Outcome | | Total score | Quality |
| --- | --- | --- | --- | --- | --- | --- | --- | --- | --- |
| Author, year | Representativeness of the samples | Sample size | Non-responders | Ascertainment of the exposure | A: study controls for age  B: control for any additional factor | Assessment of the outcome  a) Independent blind assessment.  b) Record linkage  c) Self report | Statistical test |  |  |
| Cotrell et al., 2006 (48) | - | - | - | * | - | ** | * | 4* | Moderate |
| Lau et al., 2010 (41) | * | * | * | * | ** | ** | * | 9* | High |
| Erlen et al., 2013 (40) | - | * | * | * | ** | ** | * | 8* | High |
| Wucherer et al., 2017 (50) | - | * | * | * | - | ** | * | 6* | Moderate |

| **Author, year** | **Domains** | | | | | | | | Total score | Quality |
| --- | --- | --- | --- | --- | --- | --- | --- | --- | --- | --- |
|  | Purpose  1* | Literature  2* | Design  3* | Sampling  3* | Data collection  3* | Data analysis  3* | Rigour  2* | Conclusion and implications  1* |  |  |
| While et al., 2012 (47) | * | ** | ** | ** | ** | * | * | * | 12* | High |
| Poland et al., 2014 (44) | * | ** | ** | ** | ** | ** | * | * | 13* | High |
| Smith et al., 2015 (45) | * | ** | ** | ** | ** | ** | * | * | 13* | High |
| Maidment et al., 2017 (43) | * | ** | ** | *** | ** | *** | * | * | 15* | High |

Supplementary table 4. Quality appraisal of qualitative studies included in research synthesis.

Supplementary figure 1. Risk of bias in interventional studies.

A:

| Author, year | Bias in random sequence generation | Bias in allocation concealment | Bias in blinding of participants and personnel | Bias in blinding of outcome assessment | Bias in incomplete outcome data | Bias in selective outcome  reporting |
| --- | --- | --- | --- | --- | --- | --- |
| Brodaty et al., 2009 (39) | 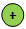 | 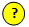 | 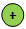 | 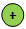 | 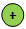 | 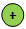 |
| Thyrian et al., 2016 (46) | 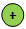 | 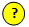 | 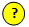 | 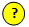 | 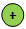 | 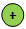 |
| Lingler et al.,  2016 (42) | 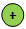 | 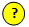 | 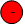 | 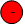 | 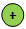 | 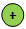 |
| 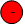 Yes (high risk of bias) 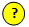 Unclear 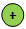 No (low risk of bias)  **RCT** | | | | | | |

B:

Supplementary figure 2**.** Risk of bias in cohort studies.

A:

| Author, year | Bias in the selection of exposed and non-exposed cohorts | Bias in the assessment of exposure | Bias in the presence of outcome of the interest at the start of the study | Bias in the control of prognostic variables (with matching or adjusting) | Bias in the assessment of the presence or absence of prognostic factors | Bias in the assessment of outcome | Bias in adequacy in the follow up of cohorts |
| --- | --- | --- | --- | --- | --- | --- | --- |
| Fiss et al.,  2013 (49) |  |  |  |  |  |  |  |
| Definitely No (low risk of bias) Probably no (Probably low risk of bias)  Definitely yes (high risk of bias) Probably Yes (Probably high risk of bias) | | | | | | | |

B:

Supplementary figure 3**.** Risk of bias in cross-sectional studies.

A:

| Author, year | Bias in the assessment of exposure | Bias in the development of the outcome of interest in case and controls | Bias in the selection of cases | Bias in the selection of controls | Bias in the control of prognostic variable |
| --- | --- | --- | --- | --- | --- |
| Cotrell et al.,  2006 (48) |  |  |  |  |  |
| Lau et al.,  2010 (41) |  |  |  |  |  |
| Erlen et al.,  2013 (40) |  |  |  |  |  |
| Wucherer et al., 2017 (50) |  |  |  |  |  |
| Definitely No (low risk of bias) Probably no (Probably low risk of bias)  Definitely yes (high risk of bias) Probably Yes (Probably high risk of bias) | | | | | |

B:
